# Supplementary material for: Socioeconomic status, depression, and incident arthritis in adults aged 50 years and over: Prospective evidence from six longitudinal aging studies
Source: PLoS One. 2025 Nov 21;20(11):e0335958. doi: 10.1371/journal.pone.0335958 (PMC12637947; doi:10.1371/journal.pone.0335958)
Supplement: S1 File — (PDF) [file pone.0335958.s001.pdf]

Supplementary Appendix

Supporting information

S1 Table. Summary of included cohorts across different regions and countries
S2 Table. Baseline characteristics of included participants by socioeconomic status
S3 Table. Baseline characteristics of included participants by depression status
S4 Table. Baseline characteristics of included participants by cohorts
S5 Table. Analyses on interaction of socioeconomic factors and depression with incident arthritis
S6 Table. Independent analysis of socioeconomic factors, depression on incident arthritis (excluding CHARLS cohort, n=63,195)
S7 Table. Independent analysis of socioeconomic factors, depression on incident arthritis (excluding ELSA cohort, n=63,497)
S8 Table. Independent analysis of socioeconomic factors, depression on incident arthritis (excluding HRS cohort, n=59,655)
S9 Table. Independent analysis of socioeconomic factors, depression on incident arthritis (excluding KLOSA cohort, n=62,839)
S10 Table. Independent analysis of socioeconomic factors, depression on incident arthritis (excluding MHAS cohort, n=58,627)
S11 Table. Independent analysis of socioeconomic factors, depression on incident arthritis (excluding SHARE cohort, n=34,412)
S12 Table. Independent analysis of socioeconomic factors, depression on incident arthritis (comparing different approaches to account for differences between countries)
S13 Table. Independent analysis of socioeconomic factors, depression on incident arthritis (missing values as a level)
S14 Table. Harmonized strategies for variables included in pooled dataset
S1 Figure. Flowchart of participant enrollment
S2 Figure. Associations between different combinations of education and wealth, and depression on incident arthritis
S3 Figure. Joint associations of education and depression with incident arthritis by age and sex
S4 Figure. Joint associations of wealth and depression with incident arthritis by age and sex
S5 Figure. Joint associations of socioeconomic status and depression with incident arthritis by age and sex
S6 Figure. Joint associations of combinations of education and wealth and depression with incident arthritis by age and sex

S1 Table. Summary of included cohorts across different regions and countries

Cohorts	Regions	Countries
CHARLS	East Asia	China
HRS	North America	United States
ELSA	Western Europe	England
KLOSA	East Asia	Korea
MHAS	North America	Mexico
SHARE	North Europe	Austria
		Belgium
		Czech Republic
		Denmark
		Estonia
		France
		Germany
		Hungary
		Italy
		Netherlands
		Poland
		Portugal
		Slovenia
		Spain
		Sweden
		Switzerland

S2 Table. Baseline characteristics of included participants by socioeconomic status

	Overall (N=68,445)	high (N=5,653)	higher-middle (N=23,378)	lower-middle (N=29,357)	low (N=10,057)
**Age at baseline, mean (SD)**	64.3 (9.7)	62.9 (8.7)	63.6 (9.1)	64.2 (9.7)	66.8 (10.8)
**Gender, N (%)**					
female	34947 (51.1)	2343 (41.4)	11242 (48.1)	15409 (52.5)	5953 (59.2)
male	33498 (48.9)	3310 (58.6)	12136 (51.9)	13948 (47.5)	4104 (40.8)
**Body mass index, N (%)**					
normal	24493 (35.8)	2434 (43.1)	8498 (36.4)	9927 (33.8)	3634 (36.1)
underweight	2725 (4.0)	88 (1.6)	706 (3.0)	1224 (4.2)	707 (7.0)
overweight	25556 (37.3)	2222 (39.3)	9136 (39.1)	10921 (37.2)	3277 (32.6)
obesity	15671 (22.9)	909 (16.1)	5038 (21.6)	7285 (24.8)	2439 (24.3)
**Marital status, N (%)**					
married/partnered	51506 (75.3)	4897 (86.6)	19180 (82.0)	21217 (72.3)	6212 (61.8)
other	16939 (24.7)	756 (13.4)	4198 (18.0)	8140 (27.7)	3845 (38.2)
**Ever smoked, N (%)**					
no	35855 (52.4)	2961 (52.4)	12040 (51.5)	15182 (51.7)	5672 (56.4)
yes	32590 (47.6)	2692 (47.6)	11338 (48.5)	14175 (48.3)	4385 (43.6)
**Ever drank, N (%)**					
no	19086 (27.9)	802 (14.2)	5199 (22.2)	9077 (30.9)	4008 (39.9)
yes	49359 (72.1)	4851 (85.8)	18179 (77.8)	20280 (69.1)	6049 (60.1)
**[a]Physical activity, N (%)**					
less than once a week of moderate or vigorous physical activity	20194 (29.5)	790 (14.0)	5367 (23.0)	9713 (33.1)	4324 (43.0)
at least once a week of moderate or vigorous physical activity	48251 (70.5)	4863 (86.0)	18011 (77.0)	19644 (66.9)	5733 (57.0)

**Depression, N (%)**					
no	48693 (71.1)	4869 (86.1)	18143 (77.6)	19930 (67.9)	5751 (57.2)
yes	19752 (28.9)	784 (13.9)	5235 (22.4)	9427 (32.1)	4306 (42.8)
**Hypertension, N (%)**					
no	41050 (60.0)	3802 (67.3)	14466 (61.9)	17125 (58.3)	5657 (56.2)
yes	27395 (40.0)	1851 (32.7)	8912 (38.1)	12232 (41.7)	4400 (43.8)
**Diabetes, N (%)**					
no	58916 (86.1)	5140 (90.9)	20594 (88.1)	24960 (85.0)	8222 (81.8)
yes	9529 (13.9)	513 (9.1)	2784 (11.9)	4397 (15.0)	1835 (18.2)
**Stroke, N (%)**					
no	65587 (95.8)	5503 (97.3)	22594 (96.6)	28016 (95.4)	9474 (94.2)
yes	2858 (4.2)	150 (2.7)	784 (3.4)	1341 (4.6)	583 (5.8)
**Cancer, N (%)**					
no	64188 (93.8)	5208 (92.1)	21804 (93.3)	27596 (94.0)	9580 (95.3)
yes	4257 (6.2)	445 (7.9)	1574 (6.7)	1761 (6.0)	477 (4.7)
**Lung disease, N (%)**					
no	64430 (94.1)	5447 (96.4)	22301 (95.4)	27498 (93.7)	9184 (91.3)
yes	4015 (5.9)	206 (3.6)	1077 (4.6)	1859 (6.3)	873 (8.7)
**Arthritis, N (%)**					
no	53969 (78.9)	4648 (82.2)	18649 (79.8)	22890 (78.0)	7782 (77.4)
yes	14476 (21.1)	1005 (17.8)	4729 (20.2)	6467 (22.0)	2275 (22.6)

Data were mean (SD) and n (%).

[a] For the MHAS cohort, physical activity was classified as "at least three times a week of moderate or vigorous physical activity" and "less than three times a week of moderate or vigorous physical activity".

S3 Table. Baseline characteristics of included participants by depression status

	Overall (N=68,445)	No-depression (N=48,693)	Depression (N=19,752)
**Age at baseline, mean (SD)**	64.3 (9.7)	64.0 (9.5)	64.8 (10.2)
**Gender, N (%)**			
female	34947 (51.1)	23128 (47.5)	11819 (59.8)
male	33498 (48.9)	25565 (52.5)	7933 (40.2)
**Body mass index, N (%)**			
normal	24493 (35.8)	17192 (35.3)	7301 (37.0)
underweight	2725 (4.0)	1412 (2.9)	1313 (6.6)
overweight	25556 (37.3)	19131 (39.3)	6425 (32.5)
obesity	15671 (22.9)	10958 (22.5)	4713 (23.9)
**Marital status, N (%)**			
married/partnered	51506 (75.3)	37788 (77.6)	13718 (69.5)
other	16939 (24.7)	10905 (22.4)	6034 (30.5)
**Ever smoked, N (%)**			
no	35855 (52.4)	24852 (51.0)	11003 (55.7)
yes	32590 (47.6)	23841 (49.0)	8749 (44.3)
**Ever drank, N (%)**			
no	19086 (27.9)	11919 (24.5)	7167 (36.3)
yes	49359 (72.1)	36774 (75.5)	12585 (63.7)
[a]**Physical activity, N (%)**			
less than once a week of moderate or vigorous physical activity	20194 (29.5)	11600 (23.8)	8594 (43.5)

at least once a week of moderate or vigorous physical activity	48251 (70.5)	37093 (76.2)	11158 (56.5)
**Education, N (%)**			
low	31881 (46.6)	19538 (40.1)	12343 (62.5)
medium	23868 (34.9)	18646 (38.3)	5222 (26.4)
high	12696 (18.5)	10509 (21.6)	2187 (11.1)
[b]**Total household wealth, N (%)**			
Q1	17119 (25.0)	10829 (22.2)	6290 (31.8)
Q2	17115 (25.0)	11970 (24.6)	5145 (26.0)
Q3	17108 (25.0)	12566 (25.8)	4542 (23.0)
Q4	17103 (25.0)	13328 (27.4)	3775 (19.1)
**Socioeconomic status, N (%)**			
high	5653 (8.3)	4869 (10.0)	784 (4.0)
higher-middle	23378 (34.2)	18143 (37.3)	5235 (26.5)
lower-middle	29357 (42.9)	19930 (40.9)	9427 (47.7)
low	10057 (14.7)	5751 (11.8)	4306 (21.8)
**Hypertension, N (%)**			
no	41050 (60.0)	30001 (61.6)	11049 (55.9)
yes	27395 (40.0)	18692 (38.4)	8703 (44.1)
**Diabetes, N (%)**			
no	58916 (86.1)	42656 (87.6)	16260 (82.3)
yes	9529 (13.9)	6037 (12.4)	3492 (17.7)
**Stroke, N (%)**			
no	65587 (95.8)	47136 (96.8)	18451 (93.4)
yes	2858 (4.2)	1557 (3.2)	1301 (6.6)
**Cancer, N (%)**			

no	64188 (93.8)	45733 (93.9)	18455 (93.4)
yes	4257 (6.2)	2960 (6.1)	1297 (6.6)
**Lung disease, N (%)**			
no	64430 (94.1)	46398 (95.3)	18032 (91.3)
yes	4015 (5.9)	2295 (4.7)	1720 (8.7)
**Arthritis, N (%)**			
no	53969 (78.9)	39032 (80.2)	14937 (75.6)
yes	14476 (21.1)	9661 (19.8)	4815 (24.4)

Data were mean (SD) and n (%).

[a] For the MHAS cohort, physical activity was classified as "at least three times a week of moderate or vigorous physical activity" and "less than three times a week of moderate or vigorous physical activity".

[b] Total household wealth, Q means quartile by a country. For example, SHARE cohort includes multiple countries, we calculated quartile by each country, summed together in one cohort and finally pooled all cohorts. Q1-Q4: from low to high.

S4 Table. Baseline characteristics of included participants by cohorts

	Overall (N=68,445)	CHARLS (N=5,250)	ELSA (N=4,948)	HRS (N=8,790)	KLOSA (N=5,606)	MHAS (N=9818)	SHARE (N=34,033)
**Age at baseline, mean (SD)**	64.3 (9.7)	61.9 (8.3)	65.0 (9.1)	62.7 (10.3)	64.6 (10.2)	63.7 (9.2)	65.0 (9.7)
**Gender, N (%)**							
female	34947 (51.1)	2449 (46.6)	2441 (49.3)	4464 (50.8)	2736 (48.8)	5259 (53.6)	17598 (51.7)
male	33498 (48.9)	2801 (53.4)	2507 (50.7)	4326 (49.2)	2870 (51.2)	4559 (46.4)	16435 (48.3)
**Body mass index, N (%)**							
normal	24493 (35.8)	2659 (50.6)	1560 (31.5)	2412 (27.4)	2496 (44.5)	2735 (27.9)	12631 (37.1)
underweight	2725 (4.0)	694 (13.2)	226 (4.6)	147 (1.7)	300 (5.4)	687 (7.0)	671 (2.0)
overweight	25556 (37.3)	1277 (24.3)	1754 (35.4)	3396 (38.6)	1608 (28.7)	3745 (38.1)	13776 (40.5)
obesity	15671 (22.9)	620 (11.8)	1408 (28.5)	2835 (32.3)	1202 (21.4)	2651 (27.0)	6955 (20.4)
**Marital status, N (%)**							
married/partnered	51506 (75.3)	4503 (85.8)	3731 (75.4)	5915 (67.3)	4536 (80.9)	7042 (71.7)	25779 (75.7)
other	16939 (24.7)	747 (14.2)	1217 (24.6)	2875 (32.7)	1070 (19.1)	2776 (28.3)	8254 (24.3)
**Ever smoked, N (%)**							
no	35855 (52.4)	2910 (55.4)	1967 (39.8)	4018 (45.7)	3590 (64.0)	5996 (61.1)	17374 (51.1)
yes	32590 (47.6)	2340 (44.6)	2981 (60.2)	4772 (54.3)	2016 (36.0)	3822 (38.9)	16659 (48.9)
**Ever drank, N (%)**							
no	19086 (27.9)	3119 (59.4)	599 (12.1)	3193 (36.3)	297 (5.3)	7307 (74.4)	4571 (13.4)
yes	49359 (72.1)	2131 (40.6)	4349 (87.9)	5597 (63.7)	5309 (94.7)	2511 (25.6)	29462 (86.6)
**[a]Physical activity, N (%)**							

less than once a week of moderate or vigorous physical activity	20194 (29.5)	2784 (53.0)	827 (16.7)	2138 (24.3)	3575 (63.8)	5755 (58.6)	5115 (15.0)
at least once a week of moderate or vigorous physical activity	48251 (70.5)	2466 (47.0)	4121 (83.3)	6652 75.7)	2031 (36.2)	4063 (41.4)	28918 (85.0)
**Education, N (%)**							
low	31881 (46.6)	4641 (88.4)	1149 (23.2)	1483 (16.9)	3241 (57.8)	8337 (84.9)	13030 (38.3)
medium	23868 (34.9)	519 (9.9)	2633 (53.2)	4994 (56.8)	1764 (31.5)	368 (3.7)	13590 (39.9)
high	12696 (18.5)	90 (1.7)	1166 (23.6)	2313 (26.3)	601 (10.7)	1113 (11.3)	7413 (21.8)
[b]**Total household wealth, N (%)**							
Q1	17119 (25.0)	1313 (25.0)	1237 (25.0)	2198 (25.0)	1402 (25.0)	2455 (25.0)	8514 (25.0)
Q2	17115 (25.0)	1313 (25.0)	1237 (25.0)	2198 (25.0)	1402 (25.0)	2455 (25.0)	8510 (25.0)
Q3	17108 (25.0)	1312 (25.0)	1237 (25.0)	2197 (25.0)	1401 (25.0)	2454 (25.0)	8507 (25.0)
Q4	17103 (25.0)	1312 (25.0)	1237 (25.0)	2197 (25.0)	1401 (25.0)	2454 (25.0)	8502 (25.0)
**Socioeconomic status, N (%)**							
high	5653 (8.3)	69 (1.3)	534 (10.8)	1086 (12.4)	310 (5.5)	574 (5.8)	3080 (9.1)
higher-middle	23378 (34.2)	1413 (26.9)	1956 (39.5)	3429 (39.0)	1748 (31.2)	2403 (24.5)	12429 (36.5)
lower-middle	29357 (42.9)	2548 (48.5)	1955 (39.5)	3609 (41.1)	2558 (45.6)	4564 (46.5)	14123 (41.5)
low	10057 (14.7)	1220 (23.2)	503 (10.2)	666 (7.6)	990 (17.7)	2277 (23.2)	4401 (12.9)
**Depression, N (%)**							
no	48693 (71.1)	2348 (44.7)	4212 (85.1)	7295 (83.0)	2812 (50.2)	6194 (63.1)	25832 (75.9)
yes	19752 (28.9)	2902 (55.3)	736 (14.9)	1495 (17.0)	2794 (49.8)	3624 (36.9)	8201 (24.1)
**Hypertension, N (%)**							
no	41050 (60.0)	3837 (73.1)	3178 (64.2)	4626 (52.6)	3733 (66.6)	5168 (52.6)	20508 (60.3)
yes	27395 (40.0)	1413 (26.9)	1770 (35.8)	4164 (47.4)	1873 (33.4)	4650 (47.4)	13525 (39.7)
**Diabetes, N (%)**							
no	58916 (86.1)	4892 (93.2)	4495 (90.8)	7268 (82.7)	4792 (85.5)	7533 (76.7)	29936 (88.0)

yes	9529 (13.9)	358 (6.8)	453 (9.2)	1522 (17.3)	814 (14.5)	2285 (23.3)	4097 (12.0)
**Stroke, N (%)**							
no	65587 (95.8)	5118 (97.5)	4797 (96.9)	8346 (94.9)	5339 (95.2)	9560 (97.4)	32427 (95.3)
yes	2858 (4.2)	132 (2.5)	151 (3.1)	444 (5.1)	267 (4.8)	258 (2.6)	1606 (4.7)
**Cancer, N (%)**							
no	64188 (93.8)	5200 (99.0)	4474 (90.4)	7941 (90.3)	5378 (95.9)	9554 (97.3)	31641 (93.0)
yes	4257 (6.2)	50 (1.0)	474 (9.6)	849 (9.7)	228 (4.1)	264 (2.7)	2392 (7.0)
**Lung disease, N (%)**							
no	64430 (94.1)	4766 (90.8)	4740 (95.8)	8382 (95.4)	5467 (97.5)	9113 (92.8)	31962 (93.9)
yes	4015 (5.9)	484 (9.2)	208 (4.2)	408 (4.6)	139 (2.5)	705 (7.2)	2071 (6.1)
**Arthritis, N (%)**							
no	53969 (78.9)	3861 (73.5)	4270 (86.3)	6412 (72.9)	5205 (92.8)	8862 (90.3)	25359 (74.5)
yes	14476 (21.1)	1389 (26.5)	678 (13.7)	2378 (27.1)	401 (7.2)	956 (9.7)	8674 (25.5)

Data were mean (SD) and n (%).

[a] For the MHAS cohort, physical activity was classified as "at least three times a week of moderate or vigorous physical activity" and "less than three times a week of moderate or vigorous physical activity".

[b] Total household wealth, Q means quartile by a country. For example, SHARE cohort includes multiple countries, we calculated quartile by each country, summed together in one cohort and finally pooled all cohorts. Q1-Q4: from low to high.

S5 Table. Analyses on interaction of socioeconomic factors and depression with incident arthritis

	No-depression	Depression	Multiplicative interaction	Additive interaction		
				RERI	AP	S
	HR (95%CI)	HR (95%CI)	HR (95%CI)	HR (95%CI)	HR (95%CI)	HR (95%CI)
**Education**						
medium and high	Ref.	1.38 (1.31-1.46)	0.96 (0.89-1.03)	-0.01 (-0.10-0.09)	0.00 (-0.07-0.06)	0.99 (0.82-1.19)
low	1.14 (1.09-1.20)	1.52 (1.44-1.60)				
**Wealth**						
more than medium	Ref.	1.35 (1.28-1.43)	1.00 (0.93-1.07)	0.04 (-0.05-0.13)	0.03 (-0.03-0.09)	1.09 (0.90-1.31)
less than medium	1.13 (1.08-1.17)	1.52 (1.45-1.60)				
**SES**						
higher-middle and above	Ref.	1.34 (1.26-1.43)	1.00 (0.93-1.08)	0.06 (-0.04-0.16)	0.04 (-0.03-0.10)	1.12 (0.92-1.35)
lower-middle and below	1.16 (1.11-1.21)	1.56 (1.48-1.64)				

All models were adjusted for age at baseline, gender, body mass index, country (strata), marital status, smoking, drinking, physical activity, prevalent hypertension, diabetes, stroke, cancer and lung disease. SES, socioeconomic status; RERI, relative excess risk due to interaction; AP, attributable proportion due to interaction; S, the synergy index; HR, hazard ratio; CI, confidence interval.

S6 Table. Independent analysis of socioeconomic factors, depression on incident arthritis (excluding CHARLS cohort, n=63,195)

	Events	Total	Model1 HR (95%CI)	Model2 HR (95%CI)	Model3 HR (95%CI)
**Education**					
high	2395	12606	Ref.	Ref.	Ref.
medium	5198	23349	1.19 (1.14-1.25)	1.16 (1.10-1.22)	1.12 (1.06-1.17)
low	5494	27240	1.49 (1.41-1.56)	1.31 (1.24-1.38)	1.22 (1.16-1.28)
**Wealth**					
fourth quartile	2989	15791	Ref.	Ref.	Ref.
third quartile	3150	15796	1.09 (1.04-1.15)	1.08 (1.02-1.13)	1.05 (1.00-1.10)
second quartile	3371	15802	1.22 (1.16-1.28)	1.18 (1.12-1.24)	1.12 (1.07-1.18)
first quartile	3577	15806	1.34 (1.28-1.41)	1.27 (1.21-1.34)	1.20 (1.14-1.26)
**Socioeconomic status**					
high	995	5584	Ref.	Ref.	Ref.
higher-middle	4431	21965	1.22 (1.14-1.30)	1.17 (1.09-1.25)	1.12 (1.05-1.20)
lower-middle	5736	26809	1.45 (1.36-1.55)	1.36 (1.27-1.46)	1.26 (1.17-1.35)
low	1925	8837	1.70 (1.57-1.83)	1.46 (1.35-1.58)	1.33 (1.22-1.44)
**Depression**					
no	9171	46345	Ref.	Ref.	Ref.
yes	3916	16850	1.49 (1.44-1.55)	1.38 (1.33-1.44)	1.33 (1.28-1.38)

Model1 was unadjusted (country as a strata term). Model2 adjusted for age at baseline, gender. Model3 further adjusted for body mass index, marital status, smoking,

drinking, physical activity, prevalent hypertension, diabetes, stroke, cancer and lung disease. HR, hazard ratios; CI, confidence interval.

S7 Table. Independent analysis of socioeconomic factors, depression on incident arthritis (excluding ELSA cohort, n=63,497)

	Events	Total	Model1 HR (95%CI)	Model2 HR (95%CI)	Model3 HR (95%CI)
**Education**					
high	2266	11530	Ref.	Ref.	Ref.
medium	4914	21235	1.18 (1.13-1.25)	1.16 (1.10-1.22)	1.12 (1.07-1.18)
low	6618	30732	1.51 (1.44-1.59)	1.34 (1.27-1.42)	1.27 (1.20-1.34)
**Wealth**					
fourth quartile	3097	15866	Ref.	Ref.	Ref.
third quartile	3347	15871	1.12 (1.06-1.17)	1.10 (1.05-1.16)	1.09 (1.03-1.14)
second quartile	3582	15878	1.24 (1.18-1.30)	1.21 (1.15-1.27)	1.17 (1.11-1.23)
first quartile	3772	15882	1.36 (1.30-1.43)	1.29 (1.23-1.36)	1.24 (1.18-1.30)
**Socioeconomic status**					
high	932	5119	Ref.	Ref.	Ref.
higher-middle	4476	21422	1.23 (1.15-1.32)	1.19 (1.11-1.28)	1.15 (1.07-1.23)
lower-middle	6186	27402	1.49 (1.39-1.60)	1.40 (1.31-1.50)	1.32 (1.23-1.41)
low	2204	9554	1.73 (1.60-1.87)	1.51 (1.39-1.63)	1.40 (1.29-1.51)
**Depression**					
no	9117	44481	Ref.	Ref.	Ref.
yes	4681	19016	1.51 (1.45-1.57)	1.41 (1.36-1.46)	1.36 (1.31-1.42)

Model1 was unadjusted (country as a strata term). Model2 adjusted for age at baseline, gender. Model3 further adjusted for body mass index, marital status, smoking,

drinking, physical activity, prevalent hypertension, diabetes, stroke, cancer and lung disease. HR, hazard ratios; CI, confidence interval.

S8 Table. Independent analysis of socioeconomic factors, depression on incident arthritis (excluding HRS cohort, n=59,655)

	Events	Total	Model1<br>HR (95%CI)	Model2<br>HR (95%CI)	Model3<br>HR (95%CI)
**Education**					
high	1858	10383	Ref.	Ref.	Ref.
medium	3880	18874	1.15 (1.09-1.22)	1.13 (1.07-1.19)	1.10 (1.04-1.17)
low	6360	30398	1.50 (1.42-1.59)	1.30 (1.23-1.37)	1.23 (1.16-1.30)
**Wealth**					
fourth quartile	2720	14906	Ref.	Ref.	Ref.
third quartile	2916	14911	1.11 (1.05-1.17)	1.09 (1.03-1.15)	1.08 (1.02-1.13)
second quartile	3160	14917	1.25 (1.18-1.31)	1.19 (1.13-1.25)	1.16 (1.10-1.22)
first quartile	3302	14921	1.36 (1.29-1.43)	1.25 (1.18-1.31)	1.20 (1.14-1.27)
**Socioeconomic status**					
high	761	4567	Ref.	Ref.	Ref.
higher-middle	3818	19949	1.20 (1.11-1.30)	1.15 (1.06-1.24)	1.12 (1.03-1.21)
lower-middle	5444	25748	1.47 (1.37-1.59)	1.34 (1.24-1.45)	1.28 (1.18-1.38)
low	2075	9391	1.71 (1.57-1.86)	1.42 (1.31-1.55)	1.34 (1.23-1.46)
**Depression**					
no	7780	41398	Ref.	Ref.	Ref.
yes	4318	18257	1.52 (1.46-1.58)	1.39 (1.34-1.45)	1.36 (1.30-1.42)

Model1 was unadjusted (country as a strata term). Model2 adjusted for age at baseline, gender. Model3 further adjusted for body mass index, marital status, smoking,

drinking, physical activity, prevalent hypertension, diabetes, stroke, cancer and lung disease. HR, hazard ratios; CI, confidence interval.

S9 Table. Independent analysis of socioeconomic factors, depression on incident arthritis (excluding KLOSA cohort, n=62,839)

			Model1	Model2	Model3
	Events	Total	HR (95%CI)	HR (95%CI)	HR (95%CI)
**Education**					
high	2395	12095	Ref.	Ref.	Ref.
medium	5202	22104	1.18 (1.13-1.24)	1.16 (1.10-1.21)	1.12 (1.06-1.17)
low	6478	28640	1.47 (1.40-1.55)	1.31 (1.24-1.38)	1.23 (1.17-1.30)
**Wealth**					
fourth quartile	3192	15702	Ref.	Ref.	Ref.
third quartile	3419	15707	1.11 (1.06-1.16)	1.10 (1.04-1.15)	1.08 (1.03-1.13)
second quartile	3648	15713	1.23 (1.17-1.29)	1.20 (1.14-1.26)	1.16 (1.10-1.21)
first quartile	3816	15717	1.34 (1.27-1.40)	1.28 (1.22-1.34)	1.22 (1.16-1.28)
**Socioeconomic status**					
high	1000	5343	Ref.	Ref.	Ref.
higher-middle	4629	21630	1.20 (1.12-1.29)	1.16 (1.08-1.24)	1.12 (1.04-1.20)
lower-middle	6280	26799	1.45 (1.35-1.55)	1.37 (1.28-1.46)	1.28 (1.20-1.37)
low	2166	9067	1.65 (1.53-1.78)	1.45 (1.34-1.56)	1.34 (1.24-1.45)
**Depression**					
no	9492	45881	Ref.	Ref.	Ref.
yes	4583	16958	1.51 (1.45-1.57)	1.41 (1.36-1.47)	1.37 (1.32-1.42)

Model1 was unadjusted (country as a strata term). Model2 adjusted for age at baseline, gender. Model3 further adjusted for body mass index, marital status, smoking, drinking, physical activity, prevalent hypertension, diabetes, stroke, cancer and lung disease. HR, hazard ratios; CI, confidence interval.

S10 Table. Independent analysis of socioeconomic factors, depression on incident arthritis (excluding MHAS cohort, n=58,627)

			Model1	Model2	Model3
	Events	Total	HR (95%CI)	HR (95%CI)	HR (95%CI)
**Education**					
high	2340	11583	Ref.	Ref.	Ref.
medium	5267	23500	1.18 (1.13-1.24)	1.15 (1.10-1.21)	1.12 (1.06-1.17)
low	5913	23544	1.49 (1.42-1.57)	1.32 (1.25-1.39)	1.24 (1.18-1.31)
**Wealth**					
fourth quartile	3030	14649	Ref.	Ref.	Ref.
third quartile	3279	14654	1.12 (1.07-1.18)	1.11 (1.06-1.16)	1.09 (1.04-1.14)
second quartile	3525	14660	1.26 (1.20-1.32)	1.22 (1.16-1.28)	1.17 (1.12-1.23)
first quartile	3686	14664	1.37 (1.30-1.44)	1.30 (1.24-1.36)	1.23 (1.17-1.29)
**Socioeconomic status**					
high	977	5079	Ref.	Ref.	Ref.
higher-middle	4491	20975	1.19 (1.11-1.27)	1.14 (1.07-1.23)	1.10 (1.03-1.18)
lower-middle	6023	24793	1.45 (1.36-1.55)	1.37 (1.28-1.46)	1.28 (1.19-1.37)
low	2029	7780	1.68 (1.56-1.82)	1.46 (1.35-1.58)	1.34 (1.24-1.45)
**Depression**					
no	9151	42499	Ref.	Ref.	Ref.
yes	4369	16128	1.50 (1.45-1.56)	1.41 (1.36-1.46)	1.37 (1.31-1.42)

Model1 was unadjusted (country as a strata term). Model2 adjusted for age at baseline, gender. Model3 further adjusted for body mass index, marital status, smoking, drinking, physical activity, prevalent hypertension, diabetes, stroke, cancer and lung disease. HR, hazard ratios; CI, confidence interval.

S11 Table. Independent analysis of socioeconomic factors, depression on incident arthritis (excluding SHARE cohort, n=34,412)

			Model1	Model2	Model3
	Events	Total	HR (95%CI)	HR (95%CI)	HR (95%CI)
**Education**					
high	801	5283	Ref.	Ref.	Ref.
medium	1974	10278	1.24 (1.14-1.34)	1.20 (1.10-1.30)	1.16 (1.06-1.26)
low	3027	18851	1.58 (1.45-1.73)	1.45 (1.32-1.59)	1.39 (1.27-1.53)
**Wealth**					
fourth quartile	1292	8601	Ref.	Ref.	Ref.
third quartile	1404	8601	1.10 (1.02-1.19)	1.09 (1.01-1.18)	1.08 (1.00-1.17)
second quartile	1514	8605	1.23 (1.14-1.32)	1.22 (1.13-1.32)	1.19 (1.11-1.29)
first quartile	1592	8605	1.32 (1.23-1.42)	1.31 (1.22-1.41)	1.27 (1.17-1.37)
**Socioeconomic status**					
high	360	2573	Ref.	Ref.	Ref.
higher-middle	1800	10949	1.26 (1.13-1.41)	1.22 (1.09-1.36)	1.18 (1.05-1.32)
lower-middle	2666	15234	1.50 (1.34-1.67)	1.44 (1.29-1.62)	1.37 (1.23-1.54)
low	976	5656	1.69 (1.49-1.91)	1.56 (1.38-1.77)	1.48 (1.30-1.68)
**Depression**					
no	3594	22861	Ref.	Ref.	Ref.

yes	2208	11551	1.54 (1.45-1.63)	1.48 (1.40-1.57)	1.44 (1.36-1.53)

Model1 was unadjusted (country as a strata term). Model2 adjusted for age at baseline, gender. Model3 further adjusted for body mass index, marital status, smoking, drinking, physical activity, prevalent hypertension, diabetes, stroke, cancer and lung disease. HR, hazard ratios; CI, confidence interval.

S12 Table. Independent analysis of socioeconomic factors, depression on incident arthritis (comparing different approaches to account for differences between countries)

	**Randon effects model**	**Multivariable Cox model**	**Stratified Cox model**
	HR（95%CI）	HR（95%CI）	HR（95%CI）
**Education**			
high	Ref.	Ref.	Ref.
medium	1.12 (1.07-1.18)	1.12 (1.07-1.18)	1.12 (1.06-1.17)
low	1.25 (1.19-1.32)	1.25 (1.19-1.32)	1.25 (1.19-1.32)
**Wealth**			
fourth quartile	Ref.	Ref.	Ref.
third quartile	1.08 (1.03-1.13)	1.08 (1.03-1.13)	1.08 (1.03-1.13)
second quartile	1.16 (1.10-1.21)	1.16 (1.10-1.21)	1.16 (1.11-1.22)
first quartile	1.22 (1.16-1.28)	1.22 (1.16-1.28)	1.23 (1.17-1.29)
**Socioeconomic status**			
high	Ref.	Ref.	Ref.
higher-middle	1.12 (1.05-1.20)	1.12 (1.05-1.20)	1.13 (1.05-1.21)
lower-middle	1.28 (1.20-1.37)	1.28 (1.20-1.37)	1.29 (1.21-1.38)
low	1.35 (1.25-1.46)	1.35 (1.25-1.46)	1.36 (1.26-1.47)
**Depression**			
no	Ref.	Ref.	Ref.
yes	1.36 (1.31-1.42)	1.36 (1.32-1.42)	1.37 (1.32-1.42)

All models were adjusted for age at baseline, gender, body mass index, marital status, smoking, drinking, physical activity, prevalent hypertension, diabetes, stroke, cancer and lung disease. HR, hazard ratios; CI, confidence interval.

S13 Table. Independent analysis of socioeconomic factors, depression on incident arthritis (missing values as a level)

	Events	Total	Model1 HR (95%CI)	Model2 HR (95%CI)	Model3 HR (95%CI)
**Education**					
high	2411	12696	Ref.	Ref.	Ref.
medium	5287	23868	1.18 (1.13-1.24)	1.16 (1.10-1.21)	1.11 (1.06-1.17)
low	6778	31881	1.50 (1.43-1.58)	1.33 (1.26-1.40)	1.25 (1.19-1.31)
**Wealth**					
fourth quartile	3264	17103	Ref.	Ref.	Ref.
third quartile	3503	17108	1.11 (1.06-1.16)	1.10 (1.04-1.15)	1.08 (1.02-1.13)
second quartile	3760	17115	1.24 (1.18-1.30)	1.20 (1.15-1.26)	1.16 (1.10-1.21)
first quartile	3949	17119	1.35 (1.29-1.42)	1.28 (1.22-1.34)	1.22 (1.17-1.28)
**Socioeconomic status**					
high	1005	5653	Ref.	Ref.	Ref.
higher-middle	4729	23378	1.21 (1.13-1.30)	1.17 (1.09-1.25)	1.12 (1.05-1.20)
lower-middle	6467	29357	1.47 (1.37-1.57)	1.38 (1.29-1.47)	1.29 (1.20-1.38)
low	2275	10057	1.69 (1.57-1.82)	1.47 (1.36-1.59)	1.36 (1.26-1.47)
**Depression**					

no	9661	48693	Ref.	Ref.	Ref.
yes	4815	19752	1.51 (1.46-1.56)	1.41 (1.36-1.46)	1.36 (1.31-1.41)

Model1 was unadjusted (country as a strata term). Model2 adjusted for age at baseline, gender. Model3 further adjusted for body mass index, marital status, smoking, drinking, physical activity, prevalent hypertension, diabetes, stroke, cancer and lung disease. HR, hazard ratios; CI, confidence interval.

S14 Table. Harmonized strategies for variables included in pooled dataset

Variables	Harmonized values	Measurements					
		SHARE	HRS	ELSA	MHAS	CHARLS	KLOSA
Education	Low	less than upper secondary					
	Medium	upper secondary and vocational training					
	High	tertiary					
Total household wealth	First quartile	the sum of all wealth components (including residence, vehicles, saving accounts, etc.) minus other debts at the couple level (the respondent and spouse, if any) in local currencies					
	Second quartile						
	Third quartile						
	Fourth quartile						
Body mass index	Normal	>= 18.5 & < 25	>= 18.5 & < 25	>= 18.5 & < 25	>= 18.5 & < 25	>= 18.5 & < 24	>= 18.5 & < 23
	Underweight	<18.5	<18.5	<18.5	<18.5	<18.5	<18.5
	Overweight	>= 24 & < 30	>= 24 & < 30	>= 24 & < 30	>= 24 & < 30	>= 24 & < 28	>= 23 & < 25
	Obesity	>=30	>=30	>=30	>=30	>=28	>=25
Marital status	Married/partnered	married, partnered	married, married (spouse absent), partnered	married, partnered	married, partnered	married, partnered	married or living with a partner, partnered

	Other	separated, divorced, widowed, never married	separated, divorced, separated/ divorced, widowed, never married	separated, divorced, widowed, never married	separated, divorced, widowed, never married	separated, divorced, widowed, never married	separated, divorced, widowed, never married
Ever smoked	Yes	the respondent reported having smoked					
	No	the respondent reported never having smoked					
Ever drank	Yes	the respondent reported having drank					
	No	the respondent reported never having drank					
Physical activity	at least once a week of moderate or vigorous physical activity	frequency of taking part in vigorous/moderate physical activity: 2.> 1 per week 3.1 per week	frequency of taking part in vigorous/moderate physical activity: 1.3+ per week 2.1-2 per week	frequency of taking part in vigorous/moderate physical activity: 2.> 1 per week 3.1 per week	whether the respondent has participated in hard physical work on average during the last two years, three or more times a week (including	the number of days of vigorous/ moderate physical activity for at least 10 minutes every week: 1-7	the number of times per week the respondent "works out" or "exercises": >=1

					sports, heavy household chores, or other physical work, et al.): 1.yes		
	less than once a week of moderate or vigorous physical activity	4.1-3 per month<br>5.hardly ever or never	3.1-3 per month<br>4.less than 1 per month<br>5.never	4.1-3 per month<br>5.hardly ever or never	0.no	0	0
Hypertension	Yes	the respondent reported having hypertension					
	no	the respondent reported having no hypertension					
Diabetes	Yes	the respondent reported having diabetes					
	no	the respondent reported having no diabetes					
Stroke	Yes	the respondent reported having stroke					
	no	the respondent reported having no stroke					
Cancer	Yes	the respondent reported having cancer					
	no	the respondent reported having no cancer					
Lung disease	Yes	the respondent reported having lung disease					
	no	the respondent reported having no lung disease					

S1 Figure. Flowchart of participant enrollment

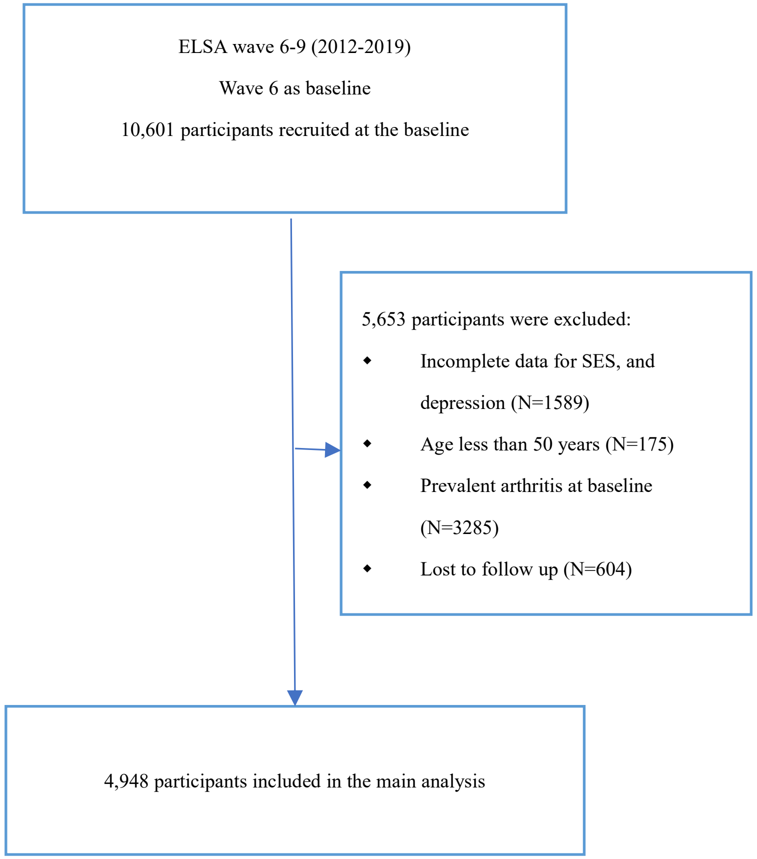
ELSA wave 6-9 (2012-2019)
Wave 6 as baseline
10,601 participants recruited at the baseline
5,653 participants were excluded:
Incomplete data for SES, and depression (N=1589)
Age less than 50 years (N=175)
Prevalent arthritis at baseline (N=3285)
Lost to follow up (N=604)
4,948 participants included in the main analysis

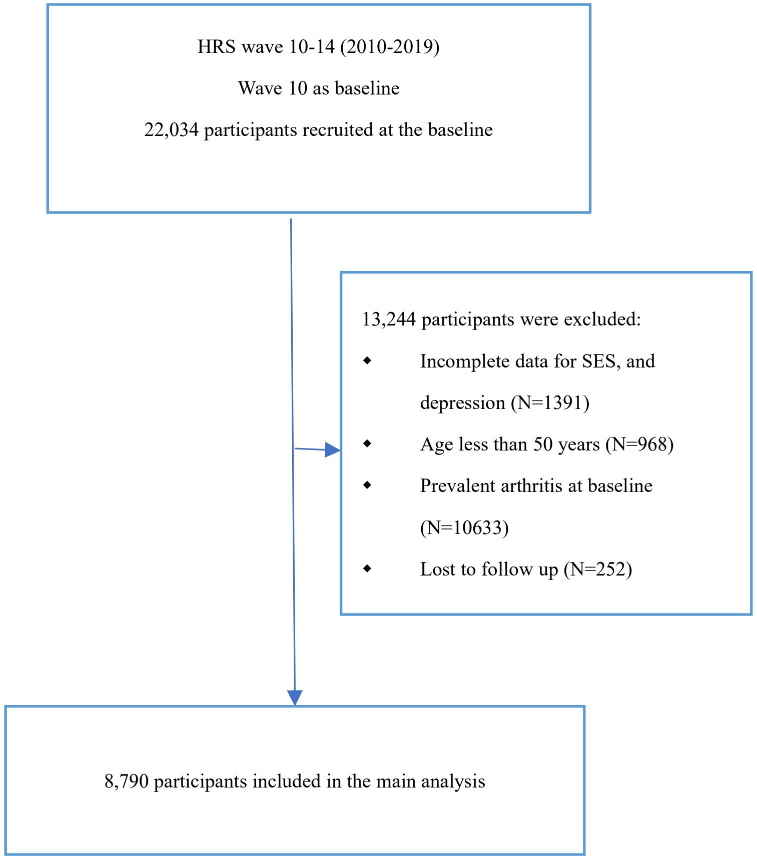
HRS wave 10-14 (2010-2019)
Wave 10 as baseline
22,034 participants recruited at the baseline
13,244 participants were excluded:
Incomplete data for SES, and depression (N=1391)
Age less than 50 years (N=968)
Prevalent arthritis at baseline (N=10633)
Lost to follow up (N=252)
8,790 participants included in the main analysis

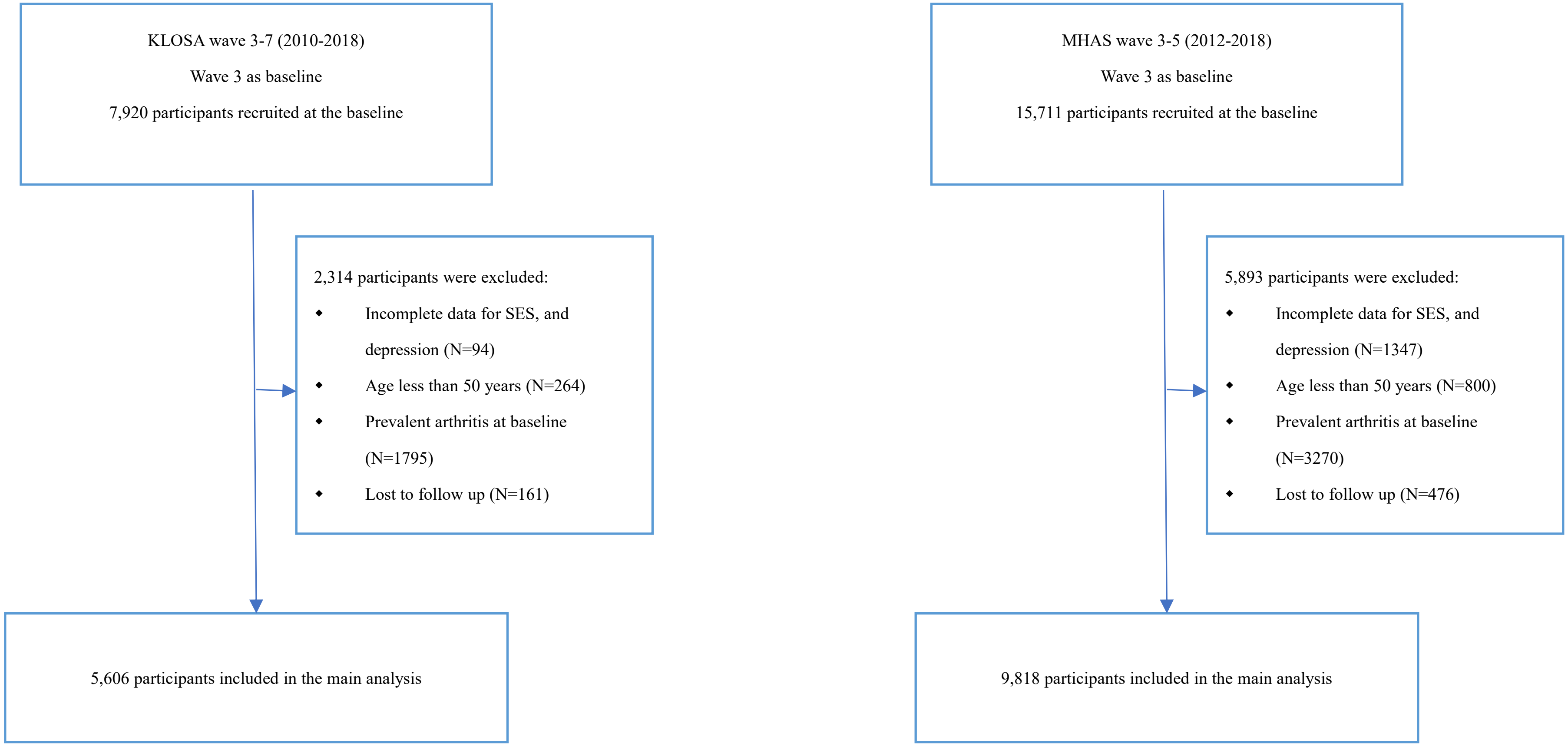
KLOSA wave 3-7 (2010-2018)
Wave 3 as baseline
7,920 participants recruited at the baseline
2,314 participants were excluded:
Incomplete data for SES, and depression (N=94)
Age less than 50 years (N=264)
Prevalent arthritis at baseline (N=1795)
Lost to follow up (N=161)
5,606 participants included in the main analysis
MHAS wave 3-5 (2012-2018)
Wave 3 as baseline
15,711 participants recruited at the baseline
5,893 participants were excluded:
Incomplete data for SES, and depression (N=1347)
Age less than 50 years (N=800)
Prevalent arthritis at baseline (N=3270)
Lost to follow up (N=476)
9,818 participants included in the main analysis

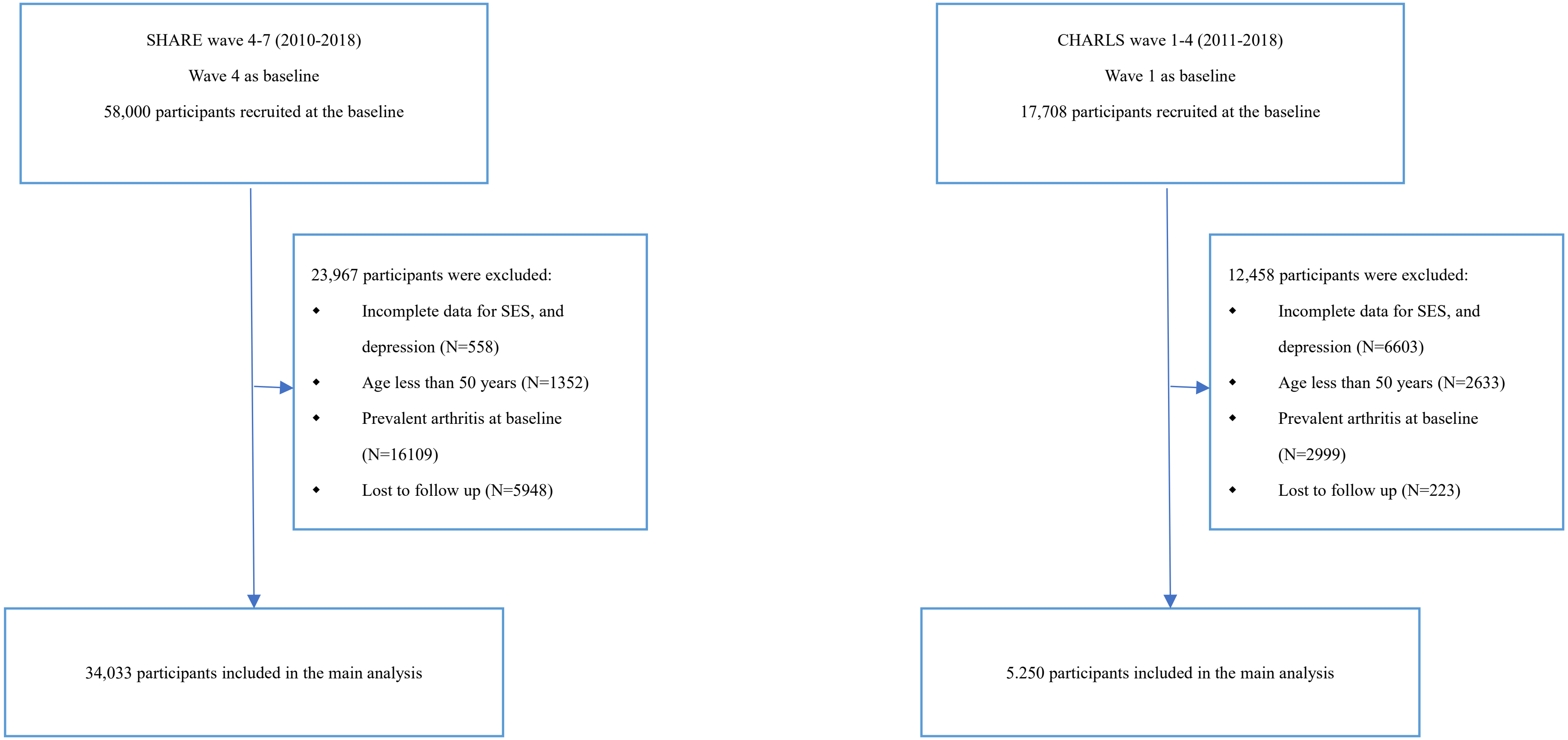

S2 Figure. Associations between different combinations of education and wealth, and depression on incident arthritis

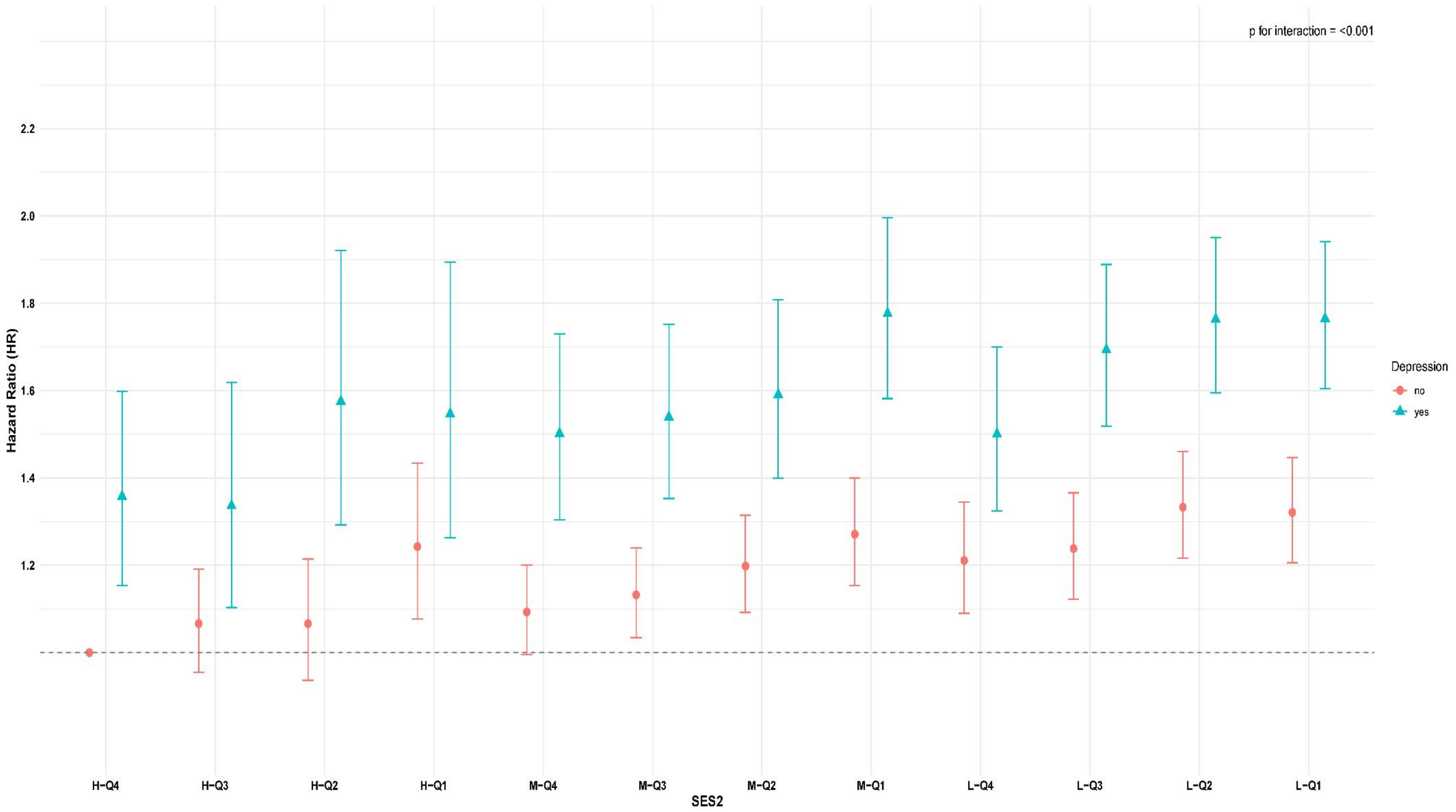

p for interaction = <0.001
2.2
2.0
1.8
1.6
1.4
1.2
Hazard Ratio (HR)
Depression
no
yes
H−Q4
H−Q3
H−Q2
H−Q1
M−Q4
M−Q3
M−Q2
M−Q1
L−Q4
L−Q3
L−Q2
L−Q1
SES2

H represented a high level of education, denoted as 'tertiary'. M represented a medium level of education, denoted as 'upper secondary and vocational training'. L represented a low level of education, denoted as ' less than upper secondary '. Q1-Q4 means quartile in total household wealth, from low to high. All models were adjusted for age at baseline, gender, body mass index, country (strata), marital status, smoking, drinking, physical activity, prevalent hypertension, diabetes, stroke, cancer and lung disease. SES, socioeconomic status; HR, hazard ratios; CI, confidence interval.

S3 Figure. Joint associations of education and depression with incident arthritis by age and sex

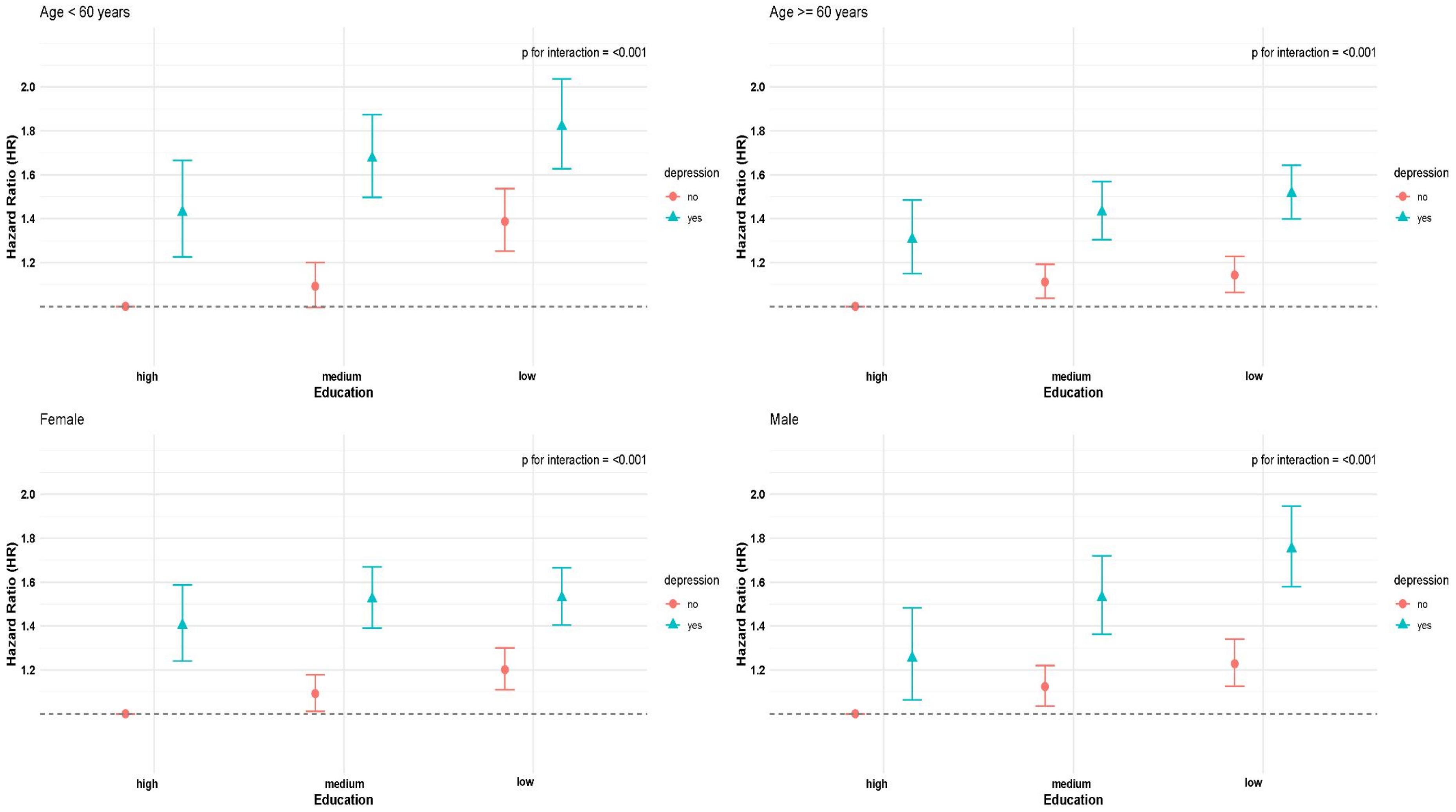

All models were adjusted for age at baseline, gender, body mass index, country (strata), marital status, smoking, drinking, physical activity, prevalent hypertension, diabetes, stroke, cancer and lung disease. HR, hazard ratios; CI, confidence interval.

S4 Figure. Joint associations of wealth and depression with incident arthritis by age and sex

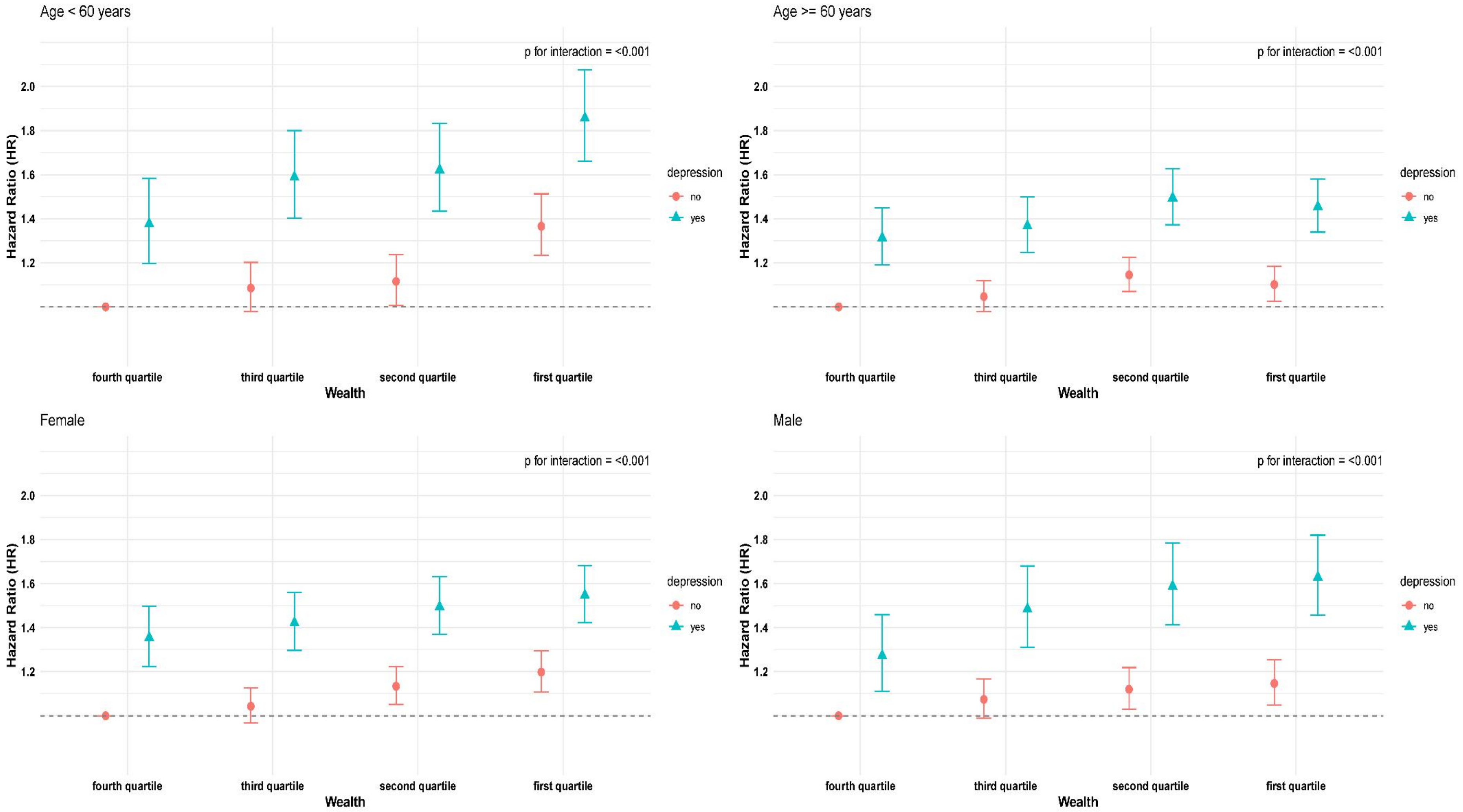

All models were adjusted for age at baseline, gender, body mass index, country (strata), marital status, smoking, drinking, physical activity, prevalent hypertension, diabetes, stroke, cancer and lung disease. HR, hazard ratios; CI, confidence interval.

S5 Figure. Joint associations of socioeconomic status and depression with incident arthritis by age and sex

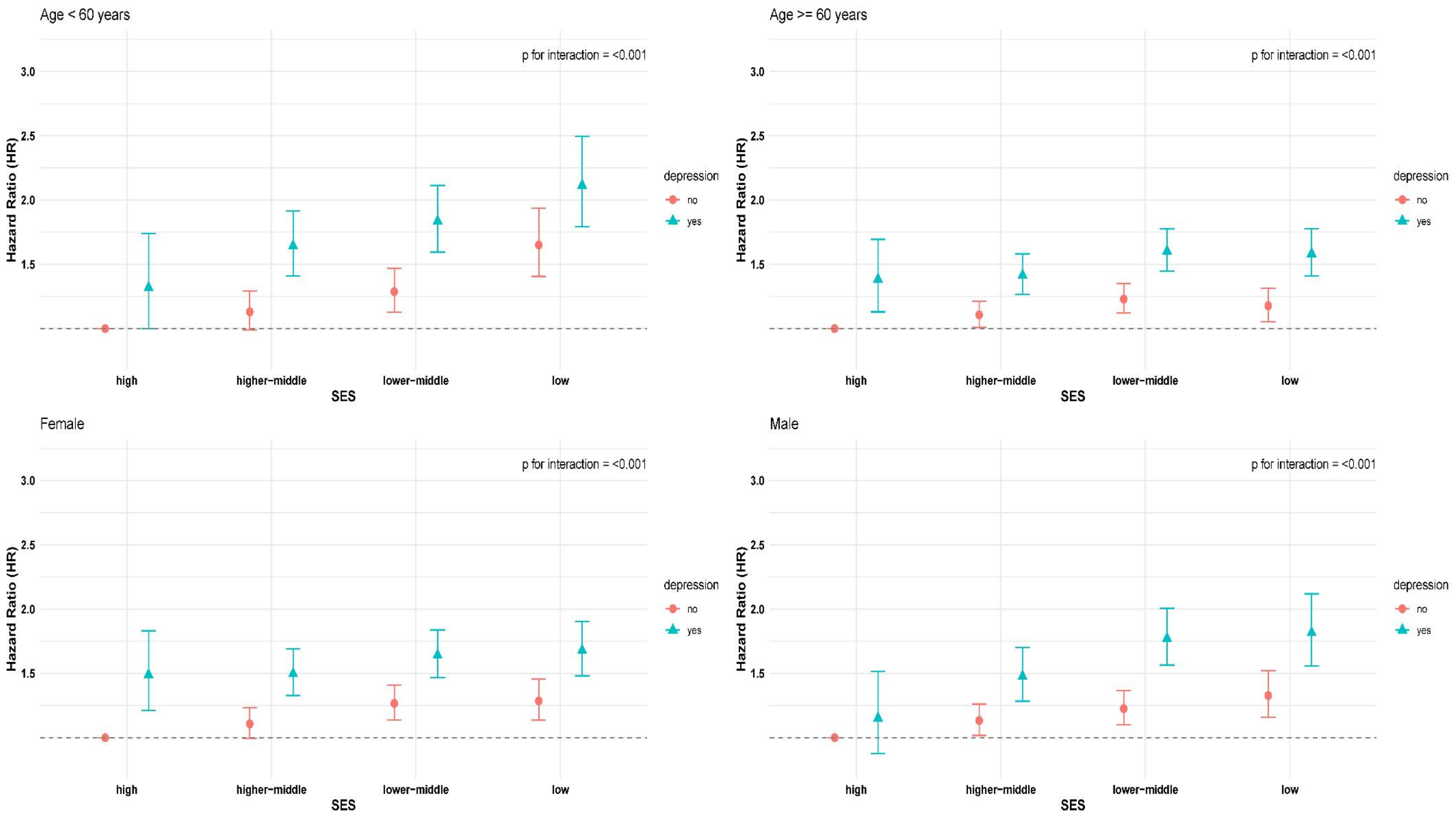

All models were adjusted for age at baseline, gender, body mass index, country (strata), marital status, smoking, drinking, physical activity, prevalent hypertension, diabetes, stroke, cancer and lung disease. HR, hazard ratios; CI, confidence interval.

S6 Figure. Joint associations of combinations of education and wealth and depression with incident arthritis by age and sex

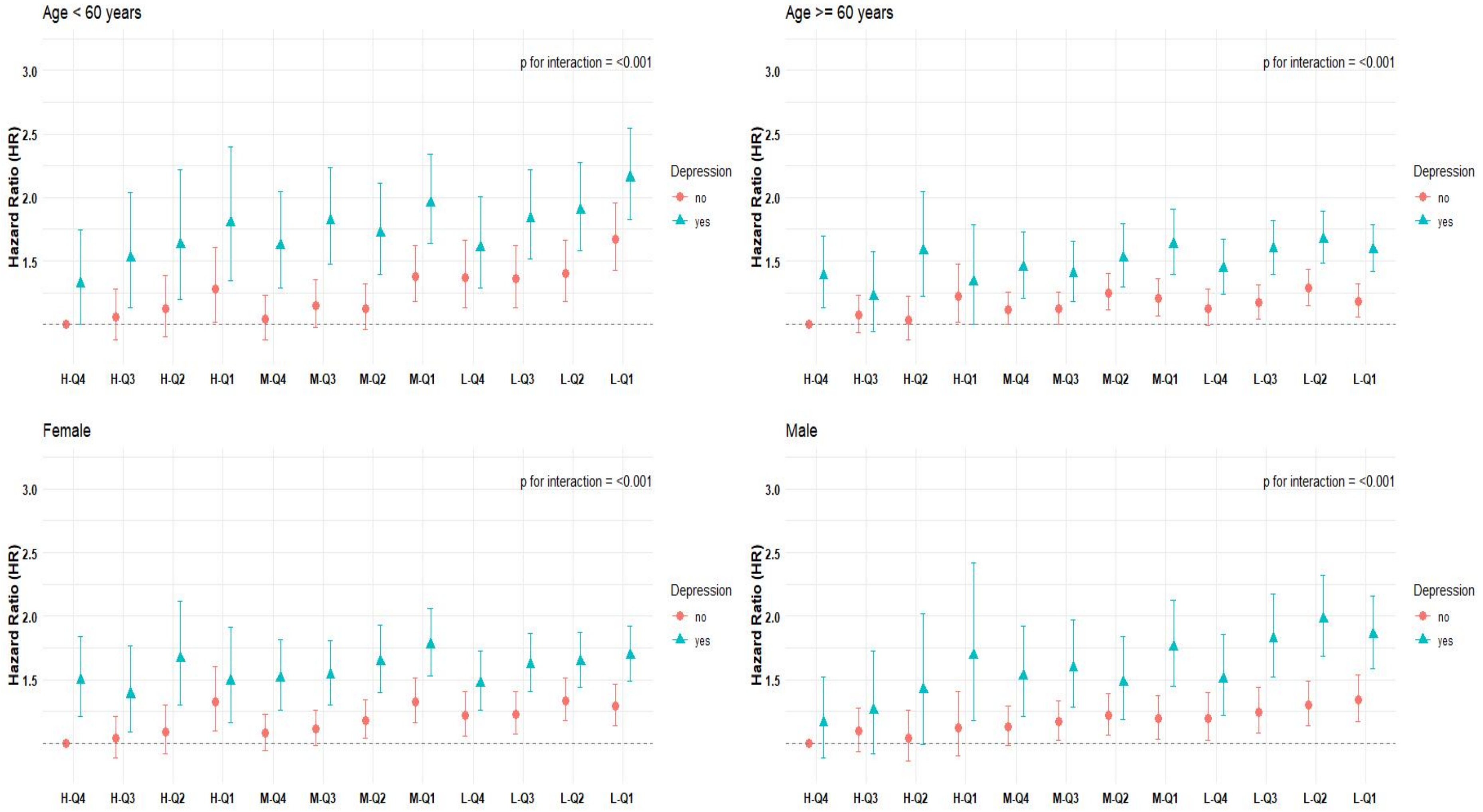

H represented a high level of education, denoted as 'tertiary'. M represented a medium level of education, denoted as 'upper secondary and vocational training'. L represented a low level of education, denoted as ' less than upper secondary '. Q1-Q4 means quartile in total household wealth, from low to high. All models were adjusted for age at baseline, gender, body mass index, country (strata), marital status, smoking, drinking, physical activity, prevalent hypertension, diabetes, stroke, cancer and lung disease. HR, hazard ratios; CI, confidence interval.
