# Supplementary material for: Socioeconomic status, depression, and incident arthritis in adults aged 50 years and over: Prospective evidence from six longitudinal aging studies
Source: PLoS One. 2025 Nov 21;20(11):e0335958. doi: 10.1371/journal.pone.0335958 (PMC12637947; doi:10.1371/journal.pone.0335958)
Supplement: Attachment — Submitted filename: Response to reviewers0916.docx [file pone.0335958.s003.docx]

To the Editor, Reviewers, PLOS ONE

Date: September 2, 2025

# Subject: Addressing Editor/Reviewer Comments for PONE-D-25-22108

Dear Editor and Reviewers,

We sincerely thank you for the constructive feedback provided by the editor and reviewers. We have carefully revised the manuscript and addressed all comments to the best of our ability. Kindly help us with your further concerns (if any) to improve our paper. Below we are providing our responses to the comments and actions that we took to address them. The responses are highlighted in blue.

Kind regards,

Authors of PONE-D-25-22108

*Socioeconomic status, depression, and incident arthritis in adults aged 50 years and over: prospective evidence from 21 countries*

PONE-D-25-22108

Socioeconomic status, depression, and incident arthritis in adults aged 50 years and over: prospective evidence from 21 countries

PLOS ONE

Dear Dr. Hu,

Thank you for submitting your manuscript to PLOS ONE. After careful consideration, we feel that it has merit but does not fully meet PLOS ONE’s publication criteria as it currently stands. Therefore, we invite you to submit a revised version of the manuscript that addresses the points raised during the review process.

Please submit your revised manuscript by Sep 15 2025 11:59PM. If you will need more time than this to complete your revisions, please reply to this message or contact the journal office at plosone@plos.org. When you're ready to submit your revision, log on to https://www.editorialmanager.com/pone/ and select the 'Submissions Needing Revision' folder to locate your manuscript file.

Please include the following items when submitting your revised manuscript:

•	A rebuttal letter that responds to each point raised by the academic editor and reviewer(s). You should upload this letter as a separate file labeled 'Response to Reviewers'.

Included

•	A marked-up copy of your manuscript that highlights changes made to the original version. You should upload this as a separate file labeled 'Revised Manuscript with Track Changes'.

Included

•	An unmarked version of your revised paper without tracked changes. You should upload this as a separate file labeled 'Manuscript'.

Included

I If you would like to make changes to your financial disclosure, please include your updated statement in your cover letter. Guidelines for resubmitting your figure files are available below the reviewer comments at the end of this letter.

If applicable, we recommend that you deposit your laboratory protocols in protocols.io to enhance the reproducibility of your results. Protocols.io assigns your protocol its own identifier (DOI) so that it can be cited independently in the future. For instructions see: https://journals.plos.org/plosone/s/submission-guidelines#loc-laboratory-protocols. Additionally, PLOS ONE offers an option for publishing peer-reviewed Lab Protocol articles, which describe protocols hosted on protocols.io. Read more information on sharing protocols at https://plos.org/protocols?utm_medium=editorial-email&utm_source=authorletters&utm_campaign=protocols.

We look forward to receiving your revised manuscript.

Kind regards,

Modupe Akintomide

Academic Editor

PLOS ONE

**Journal Requirements:**

1. When submitting your revision, we need you to address these additional requirements.

Please ensure that your manuscript meets PLOS ONE's style requirements, including those for file naming. The PLOS ONE style templates can be found at

https://journals.plos.org/plosone/s/file?id=wjVg/PLOSOne_formatting_sample_main_body.pdf and

https://journals.plos.org/plosone/s/file?id=ba62/PLOSOne_formatting_sample_title_authors_affiliations.pdf

**Response:**

We have checked the manuscript and filenames to ensure that they comply with PLOS ONE's style and naming requirements. We added “*” for corresponding author in the author list. We changed the manuscript to double-space paragraph format. Moreover, the format of all headings was also checked to meet the requirement.

2. We noticed you have some minor occurrence of overlapping text with the following previous publication(s), which needs to be addressed:

https://linkinghub.elsevier.com/retrieve/pii/S2666756823000545

https://www.thelancet.com/journals/lanhl/article/PIIS2666-7568(23)00195-2/fulltext?

In your revision ensure you cite all your sources (including your own works), and quote or rephrase any duplicated text outside the methods section. Further consideration is dependent on these concerns being addressed.

**Response:**

We thank the editors for noting this issue. We carefully reviewed the two cited publications and our manuscript. We identified minor overlapping text in the Introduction and Discussion sections, and have now rephrased all such sentences to avoid duplication. We confirm that the revised manuscript now cites all relevant sources and no unacknowledged overlapping text remains.

3. Thank you for stating the following financial disclosure:

Joint Science and Technology Funding Projects by Zunyi Bureau of Industry and

Science and Technology and the Second Affiliated Hospital of Zunyi Medical University(HZ(2024)427)

Please state what role the funders took in the study.  If the funders had no role, please state: "The funders had no role in study design, data collection and analysis, decision to publish, or preparation of the manuscript."

If this statement is not correct you must amend it as needed.

Please include this amended Role of Funder statement in your cover letter; we will change the online submission form on your behalf.

Response:

We appreciate the editor’s observation and have revised accordingly. We added the sentence “*The funders had no role in study design, data collection and analysis, decision to publish, or preparation of the manuscript.*” on the Funding section.

4. Thank you for stating the following in the Acknowledgments Section of your manuscript:

We thank all team members who have contributed to the HRS, ELSA, SHARE, KLoSA, CHARLS, and MHAS studies. This analysis used data or information from the Harmonized HRS, ELSA, SHARE, KLoSA, CHARLS, and MHAS datasets, and Codebooks developed by the Gateway to Global Aging Data, supported by the National Institute on Aging (grant numbers R01 AG030153, RC2 AG036619, and 1R03AG043052). For more information, please refer to https://g2aging.org. HRS is sponsored by the National Institute on Aging (grant number NIA U01AG009740) and is conducted by the University of Michigan. Funding for ELSA is provided by the National Institute of Aging (grants 2RO1AG7644–01A1 and 2RO1AG017644) and a consortium of UK Government departments coordinated by the Office for National Statistics. SHARE data collection has been funded by European Union (under grant agreement number 101102412) and the European Union’s Horizon 2020 research and innovation programme (under grant agreement numbers 870628 and 101015924), and various national funding sources gratefully acknowledged (https://share-eric.eu/infrastructure/funding). KLoSA is organised by the Korea Employment Information Service. CHARLS is supported by the Behavioral and Social Research division of the National Institute on Aging of the National Institute of Health (grants 1-R21-AG031372–01, 1-R01-AG037031–01, and 3-R01AG037031–03S1); the Natural Science Foundation of China (grants 70773002, 0910107022, and 71130002), the World Bank (contracts 7145915 and 7159234), and is conducted by Peking University. The Mexican Health and Aging Study is partly sponsored by the National Institutes of Health and National Institute on Aging (grant number NIH R01AG018016) in the USA and the Instituto Nacional de Estadísticay Geografía in Mexico.

We note that you have provided funding information that is not currently declared in your Funding Statement. However, funding information should not appear in the Acknowledgments section or other areas of your manuscript. We will only publish funding information present in the Funding Statement section of the online submission form.

Please remove any funding-related text from the manuscript and let us know how you would like to update your Funding Statement. Currently, your Funding Statement reads as follows:

Joint Science and Technology Funding Projects by Zunyi Bureau of Industry and

Science and Technology and the Second Affiliated Hospital of Zunyi Medical University

(HZ(2024)427)

Please include your amended statements within your cover letter; we will change the online submission form on your behalf.

Response:

We acknowledge this important point and made the suggested changes. We delete all funding-related text from the Acknowledgments section and add this to the cover letter and our Funding Statement remains unchanged. The revised Acknowledgments now read:

*“Acknowledgments*

*We thank all team members who have contributed to the HRS, ELSA, SHARE, KLoSA, CHARLS, and MHAS studies.”*

5. Thank you for uploading your study's underlying data set. Unfortunately, the repository you have noted in your Data Availability statement does not qualify as an acceptable data repository according to PLOS's standards.

At this time, please upload the minimal data set necessary to replicate your study's findings to a stable, public repository (such as figshare or Dryad) and provide us with the relevant URLs, DOIs, or accession numbers that may be used to access these data. For a list of recommended repositories and additional information on PLOS standards for data deposition, please see https://journals.plos.org/plosone/s/recommended-repositories.

Response:

Thanks for pointing it out. While we cannot directly upload the raw data to PLOS due to cohort-specific data use agreements, these datasets are fully de-identified and publicly accessible to qualified researchers via each study’s official repository. According to the other publications using the same cohorts, we changed our statement as follow:

*“**Data Availability*

*All relevant data are within the manuscript and its Supporting Information files. All data used in this study are drawn from six publicly available, harmonized longitudinal cohort studies within the Program on Global Aging, Health, and Policy. These datasets are not proprietary to the authors and are accessible to all qualified researchers through the official repositories listed below. All data are de-identified, and each study has established access procedures to protect participant confidentiality.*

*Health and Retirement Study (HRS), United States: sponsored by the National Institute on Aging and conducted by the University of Michigan. Public use datasets are available at https://hrs.isr.umich.edu/data-products.*

*Survey of Health, Ageing and Retirement in Europe (SHARE): coordinated by the Max Planck Institute. Public release datasets are available at https://share-project.org/data-access.html.*

*English Longitudinal Study of Ageing (ELSA), United Kingdom: funded by the National Institute on Aging and UK government departments. Data are available through the UK Data Service at https://discover.ukdataservice.ac.uk/series/?sn=200011.*

*Korean Longitudinal Study of Aging (KLoSA), South Korea: administered by the Korea Employment Information Service and approved by Statistics Korea. Data are available via the Korea Labor Institute at https://survey.keis.or.kr/eng/klosa/klosa01.jsp.*

*China Health and Retirement Longitudinal Study (CHARLS), China: approved by the Peking University Biomedical Ethics Review Committee. Data are available at https://charls.pku.edu.cn/en.*

*Mexican Health and Aging Study (MHAS), Mexico: sponsored by the Mexican National Institute of Statistics and Geography (INEGI), University of Texas Medical Branch, and the National Institute on Aging. Public release datasets are available at http://www.mhasweb.org”*

6. If the reviewer comments include a recommendation to cite specific previously published works, please review and evaluate these publications to determine whether they are relevant and should be cited. There is no requirement to cite these works unless the editor has indicated otherwise.

7. Please review your reference list to ensure that it is complete and correct. If you have cited papers that have been retracted, please include the rationale for doing so in the manuscript text, or remove these references and replace them with relevant current references. Any changes to the reference list should be mentioned in the rebuttal letter that accompanies your revised manuscript. If you need to cite a retracted article, indicate the article’s retracted status in the References list and also include a citation and full reference for the retraction notice.

[Note: HTML markup is below. Please do not edit.]

Reviewers' comments:

Reviewer's Responses to Questions

**Comments to the Author**

1. Is the manuscript technically sound, and do the data support the conclusions?

The manuscript must describe a technically sound piece of scientific research with data that supports the conclusions. Experiments must have been conducted rigorously, with appropriate controls, replication, and sample sizes. The conclusions must be drawn appropriately based on the data presented.

Reviewer #1: Yes

Reviewer #2: Yes

2. Has the statistical analysis been performed appropriately and rigorously?

Reviewer #1: Yes

Reviewer #2: Yes

2. Have the authors made all data underlying the findings in their manuscript fully available?

The [PLOS Data policy](http://track.editorialmanager.com/CL0/http:%2F%2Fwww.plosone.org%2Fstatic%2Fpolicies.action%23sharing/1/010f019868c27467-091716a3-3c0b-4caa-8f0a-3bab8a77bd74-000000/QmLIUCUJ1nSrZazLXZW81m7MQhTqFTQjmYSs6wpUzbg=222) requires authors to make all data underlying the findings described in their manuscript fully available without restriction, with rare exception (please refer to the Data Availability Statement in the manuscript PDF file). The data should be provided as part of the manuscript or its supporting information, or deposited to a public repository. For example, in addition to summary statistics, the data points behind means, medians and variance measures should be available. If there are restrictions on publicly sharing data—e.g. participant privacy or use of data from a third party—those must be specified.

Reviewer #1: No

Reviewer #2: Yes

**Response:**

We acknowledge this important point and made the suggested changes. However, all data underlying our analyses come from six harmonized, publicly available longitudinal cohort studies (HRS, SHARE, ELSA, KLoSA, CHARLS, and MHAS). These datasets are not proprietary to the authors; rather, they are made accessible to the international research community through well-established data access procedures that protect participant confidentiality. Researchers can freely register and download the de-identified data from each study’s official repository. In accordance with the PLOS data policy, we have clearly described the sources of data and provided links to the repositories in the Data Availability Statement. While we cannot upload the raw data directly to PLOS due to the terms of use of the cohort studies, the data are fully available to any qualified researcher without restriction beyond the standard registration process.

3. Is the manuscript presented in an intelligible fashion and written in standard English?

PLOS ONE does not copyedit accepted manuscripts, so the language in submitted articles must be clear, correct, and unambiguous. Any typographical or grammatical errors should be corrected at revision, so please note any specific errors here.

Reviewer #1: Yes

Reviewer #2: Yes

4. Review Comments to the Author

Please use the space provided to explain your answers to the questions above. You may also include additional comments for the author, including concerns about dual publication, research ethics, or publication ethics. (Please upload your review as an attachment if it exceeds 20,000 characters)

**Reviewer #1:** Socioeconomic status, depression, and incident arthritis in adults aged 50 years and over: prospective evidence from 21 countries.

The main objective of the study is clearly presented, proposing a methodological approach that addresses an existent gap in the arthritis literature, investigating the effect of depression and/or socioeconomic status (SES) on incident arthritis
Introduction
The introduction highlights the importance of examining relationship between depression and SES in the incidence of arthritis, emphasizing the need to integrate these factors into the research in order to design strategies aimed at reducing the burden of this disease.

It is true that there Is a limited number of papers that refers to depression and SES as risk factor to incident arthritis but it is well known that at least depression has a bidirectional causal relationship with arthritis (i.e Brain Behav. 2024 Jun;14(6):e3551. Consequently authors should to elaborate more about depression or/and SES can be seen as a cause of incident arthritis.

**Response:**

Thanks for pointing it out. We revised the second paragraph of introduction section for clarifying the more about the reason why depression or SES can be seen as a cause of incident arthritis as follow:

*“Socioeconomic disadvantage and depression both likely increase the risk of arthritis onset through multiple mechanisms. Lower education and limited wealth are linked to physically demanding work, adverse living conditions, unhealthy behaviors, and delayed preventive care, while depression impairs self-management and activity, disrupts sleep, and activates stress, endocrine, and immune pathways that sustain low-grade inflammation, together heightening susceptibility to incident arthritis [9–12].”*

Methods and Materials

This is a secondary analysis of six longitudinal aging studies. I think is more appropriate to refer to the six studies instead of the 21 countries.

The description of independent variables is clear enough but, please explain further if changes over time in these two variables were taken into account.

I consider adequate and pertinent the use of the various statistical methods and tests applied in the data analysis, particularly given that they were applied separately in every dataset by country(s), which allows to obtain more accurate and representative estimates tailored to each sample. In addition, it is relevant that the analysis was conducted both with and without missing data, enabling the assessment of how and to what extent the missing data may have influenced the results.

The specific ethical approvals for each cohort should be mentioned in this section. In the case of MHAS for example, the web page indicates what information is needed to include.

Response:

We appreciate these points and have revised accordingly. First, we agree that it is more accurate to refer to the six studies rather than the 21 countries, and we have revised all statements accordingly throughout the manuscript. Second, we have added the specific ethical approvals for each study as follows:

*“The Health and Retirement Study in the United States (HRS) is sponsored by the National Institute on Aging (grant number NIA U01AG009740) to the University of Michigan [18]. The Survey of Health, the Ageing and Retirement in Europe (SHARE) The SHARE study is subject to continuous ethics review by the Ethics Council of the Max Planck Society. [19]. The English Longitudinal Study of Ageing (ELSA) is founded by the National Institute of Aging (grants R01AG017644) and a consortium of UK Government departments coordinated by the Office for National Statistics [20]. The KLoSA has been approved by Statistics Korea (approval number: 33602) [21]. The China Health and Retirement Longitudinal Study (CHARLS) was approved by the Ethics Committee of Peking University (IRB approval number IRB00001052–11015) [22]. The Mexican Health and Aging Study is partly sponsored by the National Institutes of Health and National Institute on Aging (grant number NIH R01AG018016) [23]. All analyses used de-identified, publicly available data provided by these studies; no new data collection was conducted.”*

Results
The results are conclusive and present the values obtained from the applied tests and statistical models, both crude and adjusted, aimed at evaluating the extent to which various risk factors influence the development of arthritis in adults aged 50 years and older. The findings demonstrate that the study’s main objective addresses clearly and effectively explored. Specifically, depression is associated with an increased risk of developing arthritis. Therefore, significant associations are identified between several socioeconomic indicators and the incidence of the disease among participants in the longitudinal cohorts. In other words, individuals with an unfavourable socioeconomic status, low education attainment and depressive symptoms, tend to have higher risk of developing the disease. In contrast, participants with more favourable socioeconomic conditions, exhibited a lower risk, regardless of whether they reported symptoms of depression.

Discussion

The interpretation of the results is precise and valid, in the terms of the perspective of the study.

However, some issues should be considered in order to strengthen this section.

One limitation is the follow up. The variables relationship and the incidence could change if the follow up would be longer.

I also consider it essential to revisit this study from a class-based perspective. Although, the study identifies SES as a relevant variable, it does not delve into the multiple dimensions that a lower socioeconomic status entails in relation to individuals’ quality of life, nor how these conditions may contribute to an increased risk of developing arthritis.

The study adopts an approach that, while focusing on SES and depression, also centres on the level of education, under the assumption that lower educational attainment is associated with reduced knowledge of the disease and its symptoms, potentially leading to delays in diagnosis and appropriation treatment of arthritis. Moreover, it is important to emphasize that the lack of information about the disease is not exclusive to individuals with low levels of education; other fundamental aspects —such as economic access to medical services for treating chronic degenerative conditions, as well as issues related to time and distance— are also influenced by SES.

Another important aspect to highlight is that, although the data analysis by country helps to o avoid statistical bias, the proposed strategies and health policies are presented from a generalized perspective. This represents another limitation of the study, as solutions do not necessarily have to be, and often cannot be, homogenous.

Therefore, I argue that a more integral (holistic) approach that includes these diverse dimensions is essential for a truly effective approximation of the comprehension of arthritis in adults aged 50+.

**Reviewer #2:** This study is written well, easy to understand and with academic language. Nice tables, graphs, figures. I do not have revision suggestions. The impact of this study across multiple populations is helpful because we practice medicine in a global environment. And additionally similar to the recent obesity study across populations then this multi-national study helps us to better understand that many health issues are shared issues thus we can begin trying to solve the globally instead of continuing to focus only on single populations. This study can be used to justify the inclusion of international participants in studies about arthritis and social determinants of health.

Methods are well described and Results indicate that the Methods were followed.Results were presented in an understandable logical manner.

Discussion included the linkage to similar Results in other studies. Discussion included the limitations of the self reported data and self reported physician diagnosis.

Further work on identifying covariates and confounders was included.

**Response:**
We thank the reviewer for these thoughtful suggestions. We have revised the manuscript accordingly:

1. **Follow-up duration.** We acknowledge that limited follow-up may affect incidence estimates and the stability of associations. We added this to the Limitations as follow.

*“Fourth, the median follow-up duration of 5.9 years due to the data limitation. Confirmation in cohorts with longer observation windows is needed, as longer follow-up could modify both effect sizes and precision.”*

2. **Class-based SES perspective.** We agree that our study did not elaborate on the multiple dimensions through which lower socioeconomic status shapes quality of life and increases the risk of developing arthritis. However, in multi country ageing surveys that are designed for cross national harmonization, SES is most consistently operationalized with education and wealth, which ensures measurement validity at older ages and comparability across cohorts. This approach is common in high quality multi cohort analyses that jointly use ELSA and CHARLS and other HRS family studies, where individual-level SES is defined by education and total household wealth [1-2]. Our study follows this convention to preserve cross country comparability. But we agree this is a limitation, and add this to limitation section in the Discussion section as follow.

*“**Fifth, to preserve cross-country comparability, we operationalized SES using education and wealth only. Future work should incorporate additional SES dimensions related to individuals’ quality of life and delineate how these conditions contribute to an increased risk of developing arthritis.”*

1. Zhou Y, Kivimäki M, Yan LL, Carrillo-Larco RM, Zhang Y, Cheng Y, Wang H, Zhou M, Xu X. Associations between socioeconomic inequalities and progression to psychological and cognitive multimorbidities after onset of a physical condition: a multicohort study. EClinicalMedicine. 2024 Jul 25;74:102739. doi:10.1016/j.eclinm.2024.102739. PMID: 39157288; PMCID: PMC11327438.
2. Cadar D, Brocklebank L, Yan L, Zhao Y, Steptoe A. Socioeconomic and contextual differentials in memory decline: a cross-country investigation between England and China. J Gerontol B Psychol Sci Soc Sci. 2023;78(3):544-555. doi:10.1093/geronb/gbac163.

3. **Education and wealth assumption.** We agree with the reviewer that it is important to emphasize that lack of disease information is not confined to individuals with lower educational attainment. Information gaps can occur at any level of education, and other fundamental aspects of disadvantage are also shaped by SES, such as material resources, occupation, residential conditions, and access to care. To address this point, we revised the Discussion to clarify that education and wealth reflect broader, multidimensional SES constraints that influence incident arthritis risk.

“For education, lower attainment often co-occurs with constraints in material resources, occupation, residential context, and access to care, including limited income and wealth, physically demanding or insecure work, neighborhood deprivation [37,38,39]. These conditions can plausibly increase the likelihood of incident arthritis by delaying preventive and early care, increasing biomechanical load and injury risk, constraining diet and physical activity, and sustaining chronic psychosocial stress and low-grade inflammation [14,38,40]. For wealth, financial resources play a critical role in access to healthcare across the continuum from prevention to advanced treatment. Insufficient resources are associated with deferred or foregone care for chronic degenerative conditions, including constraints related to costs, the time required to obtain care, and travel distance. Lower wealth is also linked to adverse living conditions such as poorer nutrition and greater exposure to environmental risk factors, which can further predispose individuals to arthritis [39,40]. Taken together, lower SES amplifies the challenges posed by limited education and financial strain, substantially increasing the risk of developing arthritis.”

4. **Cross-country heterogeneity and policy implications.** We agree with the reviewer that a more integral and holistic perspective is essential for a full understanding of arthritis risk in adults aged 50 years and older. Accordingly, we added the specific policy section to propose context-specific strategies as follow.

“Given substantial cross-country differences in financing, benefits, service availability, workforce, geography, and social protection, implementation must be context specific. Countries with high patient cost sharing should reduce out of pocket expenses for core services and provide financial protection for essential diagnostics and medicines; systems with uneven geographic distribution of services should deploy mobile outreach, transport support, and teleconsultations; where primary care is underdeveloped, expanding standardized community protocols and establishing nurse led or physiotherapist led arthritis care pathways can improve timely treatment; where waiting times are long, increasing community rehabilitation capacity and introducing advanced practice roles can relieve bottlenecks. Aligning common clinical aims with country specific delivery offers an available path to earlier diagnosis, better symptom control, and more equitable outcomes for older adults, particularly those with lower education or limited wealth.”

We hope that the revisions have adequately addressed all concerns and that the manuscript is now suitable for publication in PLOS ONE.

5. PLOS authors have the option to publish the peer review history of their article ([what does this mean?](https://track.editorialmanager.com/CL0/https:%2F%2Fjournals.plos.org%2Fplosone%2Fs%2Feditorial-and-peer-review-process%23loc-peer-review-history/1/010f019868c27467-091716a3-3c0b-4caa-8f0a-3bab8a77bd74-000000/_PVfOGTgzdzw1fP0dg6k2oxa5MQscR3nSWpZ2_x9vVo=222)). If published, this will include your full peer review and any attached files.

If you choose “no”, your identity will remain anonymous but your review may still be made public.

**Do you want your identity to be public for this peer review?** For information about this choice, including consent withdrawal, please see our [Privacy Policy](https://track.editorialmanager.com/CL0/https:%2F%2Fwww.plos.org%2Fprivacy-policy/1/010f019868c27467-091716a3-3c0b-4caa-8f0a-3bab8a77bd74-000000/LziFQa6MKbgb8U6EkssqwGQ-FE1BhfYLSIfnEsm1WN0=222).

Reviewer #1: No

Reviewer #2: **Yes:** Jeananne Elkins MPH, DPT, PhD

[NOTE: If reviewer comments were submitted as an attachment file, they will be attached to this email and accessible via the submission site. Please log into your account, locate the manuscript record, and check for the action link "View Attachments". If this link does not appear, there are no attachment files.]

While revising your submission, please upload your figure files to the Preflight Analysis and Conversion Engine (PACE) digital diagnostic tool, [https://pacev2.apexcovantage.com/](https://track.editorialmanager.com/CL0/https:%2F%2Fpacev2.apexcovantage.com%2F/1/010f019868c27467-091716a3-3c0b-4caa-8f0a-3bab8a77bd74-000000/fA6VyjdnJQPJV4rgueHuJ1wWYvkhnL8q8ji5ExDJpq8=222). PACE helps ensure that figures meet PLOS requirements. To use PACE, you must first register as a user. Registration is free. Then, login and navigate to the UPLOAD tab, where you will find detailed instructions on how to use the tool. If you encounter any issues or have any questions when using PACE, please email PLOS at [figures@plos.org](mailto:figures@plos.org). Please note that Supporting Information files do not need this step.
